# Retracted: Overexpression of Bacterial *mtlD* Gene in Peanut Improves Drought Tolerance through Accumulation of Mannitol

**DOI:** 10.1155/2019/2434085

**Published:** 2019-12-13

**Authors:** The Scientific World Journal

*The Scientific World Journal* has retracted the article titled “Overexpression of Bacterial *mtlD* Gene in Peanut Improves Drought Tolerance through Accumulation of Mannitol” [1]. As raised on PubPeer, there is image duplication in Figure 2. Further issues were found with the figures and reporting, including when compared with the thesis of the first author [2].

The authors responded and provided some of the original figures and data, which are included as supplementary materials. The authors offered to correct the figures, and they do not agree with retraction. However, their response did not satisfy our concerns and the Editorial Board recommended retraction. A summary of the major concerns is as follows:

1. Two of the panels in each of Figures 2(e) and 2(f) are identical, i.e., panel 3 of Figure 2(e) is the same as panel 1 of Figure 2(f) and panel 4 of Figure 2(e) is the same as panel 3 of Figure 2(f). The authors say the figure duplication was inadvertent.
2. The same figure duplication also appeared in Figure 4.3 in the thesis. However, the left-hand panels in Figure 4.3A (thesis) and Figure 2(e) (article) are not the same. The authors disputed this and provided a figure they say was from the thesis, which they say shows the left-hand panels in Figure 2(e) are the same as those in Figure 4.3A in the thesis, but the thesis does not show the same image as the authors provided to us.
3. The distribution of insignificant digits (i.e., digits unimportant to the measured value) is expected to be uniform [3]. The rightmost values in the data underlying Table 3 have a significantly nonuniform distribution.
4. Other concerns include the following:
5. Figures 2(a) and 2(b) are the same: both show “deembryonated cotyledons cocultured with Agrobacterium strain LBA4404,” but the same view is shown twice. In the thesis, Figures 4.2A, C, and D show the same images as Figures 2(a), 2(c), and 2(d) in the article, but Figure 4.2B is different from Figure 2(b) in the article. Figure 4.2B in the thesis should have been used instead of the image in Figure 2(b) in the article.
6. Figure 3(d) shows “detection of mtlD gene transcription in transgenic plants using RT-PCR. Lane *N* = nontransformed line, lanes 1–8 = transgenic lines (MTD1 to MTD8), bottom row: 18SrRNA as internal control.” The left-most seven lanes (including the marker lane) are the same as those in Figure 4.33 in the thesis, which reports “RT-PCR analysis for the T2 transgenic plants before and after stress treatment,” showing six strains before and after stress. There is also undeclared splicing in Figure 3(d) between lanes 6 and 7. The authors said the RT-PCR assay in Figure 3(d) only shows the amplification after stress because there is no difference compared to before stress as mtlD is driven by the constitutive CaMV35S promoter. Since there were no significant changes in the expression level, they provided representative images of those eight lines to show the transgene was expressed when RNA was isolated and amplified following the RT-PCR protocol. To show the eight lanes along with the nontransformed line, they cut and pasted the images as one picture; they provided the original unaltered images.
7. The strain numbering and results in Table 4.24 in the thesis do not match those reported in the article in Table 3. The authors explained that they initially had 193 plants of mtlD peanut transgenics, out of which only 10 were confirmed positive. For publication, only 8 transgenic lines were taken for further analysis and, for the convenience of readers, the plants were numbered as 1–8.
8. A previous study by the authors also on mtlD in peanuts was cited as reference 8 [4], but was not discussed in detail. The authors explained that this *Australian Journal of Crop Science* article studied plants under salinity- and PEG-induced stress in 2010-11, in tissue culture for PEG-induced stress and using hydroponics for NaCl-induced stress. The present study investigated plants under water-deficit stress, including the tolerance of soil-planted transgenic lines in pots. The studies used the same transgenic lines, but the sets of plants were different.
9. Figure 3(b) was later reproduced as Figure 2(b) in Bala et al., in the *Turkish Journal of Biology* [5]. Though both show NptII amplification, the studies involve different transgenic plasmids. This was attributed to an inadvertent error and was corrected in May 2019 [6].

## Supplementary Materials

Supplementary Materials The authors responded and provided some of the original figures and data, which are included as supplementary materials.
